# Effect of Matching Between the Adopted Corporate Response Strategy and the Type of Hypocrisy Manifestation on Consumer Behavior: Mediating Role of Negative Emotions

**DOI:** 10.3389/fpsyg.2022.831197

**Published:** 2022-05-20

**Authors:** Zhigang Wang, Xintao Liu, Lei Zhang, Chao Wang, Rui Liu

**Affiliations:** ^1^Economics and Management School of Wuhan Sports University, Wuhan, China; ^2^China Baowu Iron and Steel Group Co., Ltd., Wuhan, China

**Keywords:** situational crisis communication theory, corporate hypocrisy, response strategies, negative emotions, negative behaviors

## Abstract

Consumers may sense hypocrisy in corporate social responsibility (CSR) if they note inconsistency in enterprises’ words and deeds related to CSR. This inconsistency originates from the intentional selfish actions and unintentional actions of enterprises. Studies have revealed that consumers’ perception of hypocrisy has a negative influence on enterprise operation. However, studies have not examined how corporate responses to consumers’ hypocrisy perception affect consumers’ attitude and behavior. Therefore, the present study attempted to determine the measures that should be undertaken by enterprises to reduce consumers’ negative response to them when consumers perceive them to be hypocritical. We conducted a situational simulation experiment to explore the effect of the match between corporate hypocrisy manifestation (moral hypocrisy vs. behavioral hypocrisy) and the corporate response strategy (reactive CSR communication vs. proactive CSR communication) on consumers’ negative behaviors toward an enterprise and to test the mechanism influencing this effect. The results indicated that the interaction between the type of corporate hypocrisy and the corporate response strategy has a significant effect on consumers’ negative behaviors toward an enterprise. Consumers’ negative emotions have a mediating influence on the aforementioned effect. This study explored the response strategies of enterprises during a corporate hypocrisy crisis, classified corporate hypocrisy crises into two types (moral hypocrisy vs. behavioral hypocrisy) according to the different manifestations of corporate hypocrisy, and introduced situational crisis communication theory (SCCT) into research on corporate hypocrisy. The present results help expand knowledge on corporate hypocrisy.

## Introduction

Corporate social responsibility (CSR), which is a key concern related to corporate strategic management, has been receiving increasing attention from researchers on and practitioners of corporate governance (George, 2010). Big data not only drives the development of enterprises (Kovacova et al., 2020; Kovacova and Lewis, 2021; Malkawi and Khayrullina, 2021) but also helps consumers better understand the behavior of enterprises, enhances their perceptions of enterprises, and increases the strength of consumers’ ethical consumption consciousness. CSR influences consumer behavior and is an essential component of corporate strategies; moreover, it can positively influence stakeholders, thus stimulating support for the firm (Reverte et al., 2015). CSR can thus help a firm establish a suitable image and improve its performance (Jeffrey et al., 2019). Up to 90% of the global top 500 enterprises are reported to have explicit investment in CSR (Bai and Chang, 2015). However, in practice, not all CSR activities of enterprises lead to positive consumer responses. Some CSR activities, if conducted inappropriately, have a negative effect on corporate image and cause consumers to resist the enterprise and its products (Fassin and Buelens, 2011). Consumers may respond negatively to CSR activities if they consider CSR activities to be hypocritical because they perceive inconsistency between the words and deeds of enterprises. Since Wagner et al. (2009) proposed the concept of corporate hypocrisy, the corporate hypocrisy associated with CSR has become a crucial branch of CSR research that has attracted considerable research attention (Antonetti et al., 2019; Shim and Kim, 2021). Studies have explored the meaning of corporate hypocrisy (Wagner et al., 2009), the formation of consumers’ hypocrisy perception toward CSR (Wang and Zhu, 2020), the effect of corporate hypocrisy perception on consumer behavior and attitudes, and the mechanism of this effect (Wagner et al., 2009; Wang and Li, 2015; Wang et al., 2020; Wang and Zhu, 2020). However, studies have not investigated the measures that should be undertaken by firms to reduce the negative effect on firms of consumers’ hypocrisy perception toward the CSR of these firms and the effects of these measures on consumer behavior. The present study attempted to fill the aforementioned research gap by examining the effects of corporate response strategies on consumer behavior when consumers perceive CSR to be hypocritical. We examined how consumers respond to corporate countermeasures in response to consumers’ perceiving CSR activities as hypocritical and how firms can effectively reduce the effects of such a perception on firm performance. This study extends research on corporate hypocrisy by analyzing consumers’ reactions to corporate response strategies when they perceive corporate hypocrisy.

Some examples of firms deemed to act hypocritically are provided in this section. Zhenhai Industrial—a Chinese company that manufactures men’s products and is located in Shanghai—employed a former Japanese adult video actress as its spokesperson for a youth public welfare activity in 2018. Because of this inappropriate behavior, the company was fined by the Chinese administrative department and strongly criticized by the public. To eliminate the negative publicity caused by the aforementioned incident, Zhenhai Industrial issued an apology; however, this statement did not earn it the public’s forgiveness. Dali Group—a Chinese food corporation—launched a public welfare campaign to donate a portion of its sales from 2017 to 2018 to the maintenance and development of intangible cultural heritage. However, this firm did not collect funds for its campaign, which was considered to be inconsistent with its messaging in relevant publicity. Therefore, the firm was fined by the Chinese market supervision department. To eliminate the negative impact of the aforementioned event, the firm admitted its mistakes in the welfare campaign and safeguarded its rights through judicial means. However, the response of the firm was not accepted by consumers. Lianjia—a Chinese real estate service platform—launched a welfare campaign to protect lost children in 2016. It claimed that lost children could visit any of its 6,000 chain stores to seek protection. However, the campaign was questioned by the police, who believed that lost children should ask the police for help first rather than visiting stores of Lianjia because Lianjia was unqualified for rescuing lost children. To address the doubts of the public, Lianjia explained why they launched the campaign in the media but did not employ further effective steps to address the problem. Most firms have uncertainties regarding appropriate handling of crises resulting from corporate hypocrisy, and even researchers have not examined this matter comprehensively.

Certain CSR activities might lead to consumers perceiving the firm to be hypocritical. If firms do not employ effective measures to eliminate the negative effects of consumers’ perception of firm hypocrisy, they would be unable to achieve the aims of CSR strategies. Thus, firms might hesitate to invest in CSR activities and reduce their participation in CSR.

Studies have indicated that the implementation of a CSR communication strategy can help enterprises handle a crisis (Klein and Dawar, 2004; Luo and Bhattacharya, 2006; Chernev and Blair, 2015). However, studies have not examined whether the implementation of a CSR communication strategy can help a firm handle a crisis triggered by perceived hypocrisy in the firm’s CSR. A suitable theoretical framework must be developed to examine whether a specific CSR communication strategy can be used by enterprises to mitigate the negative effects of their CSR activities being perceived as hypocritical. The success of a firm’s response to its CSR activities being perceived as hypocritical affects its willingness to continue to invest in CSR activities.

CSR communication strategies can be divided into two types: proactive and reactive CSR communication strategies (Murray and Vogel, 1997; Chen et al., 2019). The proactive type refers to strategies in which enterprises undertake CSR to establish a positive image of themselves before any negative social responsibility information is exposed about them. The reactive type refers to strategies in which enterprises attempt to repair their image after a crisis by declaring their intention to engage in CSR (Murray and Vogel, 1997). On the basis of the context of corporate hypocrisy–related crises, we define a proactive CSR communication strategy as one in which firms engage in CSR activities—to establish a trustworthy and suitable social image—long before the occurrence of a hypocrisy-related crisis. A reactive CSR communication strategy is defined as one in which firms declare their intention to engage in CSR activities after the occurrence of a corporate hypocrisy–related crisis to overcome the crisis and obtain the understanding of consumers. Situational crisis communication theory (SCCT), which was proposed by Coombs, emphasizes that the nature of a crisis should be understood before selecting a crisis communication strategy (Coombs and Holladay, 2001; Coombs, 2007). The nature of a corporate crisis depends on consumers’ perception of the degree of responsibility that the enterprise should bear for the crisis and the moral performance of the enterprise during the crisis (Coombs and Holladay, 2001; Coombs, 2007). During a hypocrisy-related crisis, consumers can evaluate the moral problems in enterprises from various perspectives. Consumers may perceive some mistakes by enterprises to be intentional and other mistakes by enterprises to be unintentional. On the basis of this information, Wagner et al. (2020) divided corporate hypocrisy into moral hypocrisy and behavioral hypocrisy. Moral hypocrisy is caused by the intentional deceptive behavior of an enterprise and originates from the selfish motivation of the enterprise. Behavior hypocrisy is caused by the unintentional inconsistent behavior of an enterprise, which is unrelated to morality (Wagner et al., 2020). According to SCCT, appropriate communication strategies should be selected according to the attribution category of a crisis (Coombs and Holladay, 2001). When an enterprise faces moral problems, a hypocrisy-related crisis faced by it might reveal information that is inconsistent with its CSR communication strategy, which can strengthen consumers’ perception of corporate hypocrisy. When an enterprise does not face moral problems, consumers do not blame the enterprise at the moral level, and a suitable CSR communication strategy might resolve a hypocrisy-related crisis faced by the enterprise. Thus, consumers’ moral evaluation of an enterprise affects their assessment of the motivation of the CSR communication strategy adopted by the enterprise and consequently their final behavior and attitude toward the enterprise.

Therefore, on the basis of SCCT and by using a scenario simulation experiment, we analyzed the effects of consumer perception of a firm on the effectiveness of various CSR communication strategies adopted by the firm in different types of hypocrisy crises. By examining firms’ strategies in response to consumers’ perception of hypocrisy in firms’ CSR, the present study deepens the existing research on corporate hypocrisy. The results of this study provide a reference for enterprises to handle hypocrisy-related crises appropriately.

The contributions of this research are as follows. First, it deepens existing research on corporate hypocrisy by investigating how firms respond when consumers perceive the CSR activities of firms to be hypocritical and how consumers react to different response strategies adopted by firms when they consider firms’ CSR activities to be hypocritical. Second, it introduces the concept of corporate hypocrisy into the field of corporate crisis management. Third, this study introduces a new theoretical perspective regarding corporate hypocrisy by applying SCCT. Finally, it expands the application scope of SCCT by applying this theory to the context of corporate hypocrisy and finding the effect of a match between a crisis situation and a firm’s response strategy on consumer behavior.

The main findings of this study are as follows. Different corporate response strategies have distinct effects on consumers’ negative behaviors toward a firm when consumers perceive corporate hypocrisy, and consumers’ negative emotions have a mediating influence on these effects. Therefore, the response strategy adopted by a firm should match the type of corporate hypocrisy-related crisis.

The remainder of this paper is organized as follows. Literature Review and Research Hypothesis section presents the relevant literature and research hypotheses. Research Design section describes the research methodology. Data Analysis section describes the analysis of the collected data. Finally, Discussion and Conclusion section presents a discussion of the results and the conclusion of this study.

## Literature Review and Research Hypothesis

### Corporate Hypocrisy

The theoretical origin of the concept of hypocrisy can be traced back to the fields of philosophy and psychology. Researchers initially introduced the concept of personal hypocrisy, with a person being regarded as a hypocrite if their words and deeds conflicted with each other (Bloomfield, 2018). A sense of hypocrisy arises because a person’s initial statement sets a standard of behavior, which is violated by subsequent behavior (Effron et al., 2018a). If the violation is considered intentional, the perceived hypocrisy is regarded as a moral transgression (Chvaja et al., 2020). The concept of hypocrisy was gradually introduced into the fields of management, organizational behavior (Carlos and Lewis, 2018), and business theory (Marín et al., 2016). Wagner et al. (2009) formally proposed the concept of corporate hypocrisy from the perspective of cognitive psychology. According to the aforementioned authors, the perception of corporate hypocrisy occurs when the CSR plan publicized by an enterprise is inconsistent with its actual action. Scholars have defined the concept of corporate hypocrisy from diverse perspectives. For example, according to Christensen et al. (2010), the perception of corporate hypocrisy occurs when an enterprise falsely projects its public behavior to be in line with its private behavior. According to Scheidler et al. (2019), corporate hypocrisy is a situation in which a firm’s behavior is inconsistent with its social identity. Yin et al. (2016) considered corporate hypocrisy to be an act of “saying one thing and doing another thing” when enterprises fulfill their social responsibilities.

The perception of corporate hypocrisy involves a psychological mechanism, and studies have examined the causes and consequences of this psychological mechanism. Research has suggested various factors that can affect the formation of consumers’ perception of corporate hypocrisy; for example, CSR implementation, consumer attribution (Wang et al., 2018), consumer perception of CSR (Kim et al., 2015), corporate reputation (Shim and Yang, 2016), CSR information framework (Shim et al., 2017), CSR information display (Scheidler et al., 2019), perception of CSR authenticity (Guèvremont and Grohmann, 2018), corporate information transparency (Losada-Otálora and Alkire, 2021), and information inconsistency (Shao and Liu, 2019). The perception of corporate hypocrisy by stakeholders negatively affects their evaluations of CSR belief, corporate attitude, and corporate reputation (Wagner et al., 2009; Arli et al., 2017) and deepens the perception of corporate egoism (Marín et al., 2016). Moreover, the aforementioned perception causes consumers to experience negative emotions and engage in negative behaviors toward enterprises (Wang et al., 2020; Wang and Zhu, 2020), reduces consumers’ willingness to buy corporate products (Guèvremont and Grohmann, 2018), and enhances consumers’ intentions to punish enterprises perceived as being hypocritical (Deng and Xu, 2017). Hypocrisy perception can also lead to employees’ emotional exhaustion and increase their turnover intention (Greenbaum et al., 2015; Scheidler et al., 2019). Factors such as consumer CSR belief (Arli et al., 2017), consumer attribution (Fan and Tian, 2017), negative consumer emotions (Wang et al., 2018), and consumer suspicion (Arli et al., 2019) influence the formation of consumers’ perception of corporate hypocrisy and the effects of this perception on firms.

The concept of corporate hypocrisy is vague, with the original definition being insufficiently interpretive, and several aspects related to corporate hypocrisy remain to be explored (Monin and Merritt, 2011; Laurent et al., 2014). Moreover, studies on corporate hypocrisy have not investigated the problem of intentionality, which refers to people confusing hypocrisy originating from the intentional behaviors of enterprises with hypocrisy originating from the unintentional behaviors of enterprises (Wagner et al., 2020). Therefore, Wagner et al. (2020) used contemporary social psychology theory to define the perception of corporate hypocrisy from three perspectives: moral hypocrisy, behavioral hypocrisy, and hypocrisy attribution. These perspectives originate from two conceptual routes: one driven by corporate deception and the other driven by purely inconsistent behavior (Wagner et al., 2020). The aforementioned definitions are clearer than those originally proposed by Wagner et al. (2009) and many other scholars. In addition, perceptions of corporate hypocrisy drive the cognitive, emotional, and behavioral responses of stakeholders (Wagner et al., 2020).

Moral hypocrisy refers to a scenario in which enterprises pretend to be more noble than they actually are. Such hypocrisy stems from the ulterior and selfish motives and deceptive behaviors of enterprises (Monin and Merritt, 2011; Effron et al., 2018b). A perception of moral hypocrisy occurs when an enterprise makes a statement regarding its moral essence, belief system, or values but does not engage in suitable behaviors to adhere to this statement (Lin and Miller, 2021). For example, Volkswagen claimed to value environmental protection but then cheated on emission requirements (Lyon and Montgomery, 2013), and Wells Fargo claimed to act ethically but secretly deceived its customers (Witman, 2018). Behavioral hypocrisy, which indicates that the behavior of an enterprise deviates from its public statements, reflects the lack of consistency between the words and deeds of an enterprise. The root of behavioral hypocrisy lies in the contradiction between words and deeds and is not based on any specific intention of an enterprise nor related to moral evaluation. This behavior does not indicate that the enterprise is malicious; however, it indicates to consumers or other stakeholders that the enterprise is unreliable, or at least unpredictable. Behavioral hypocrisy can take the form of an enterprise making ambitious commitments to stakeholders but then failing to meet expectations (Christensen et al., 2013). Hypocrisy attribution is different from moral hypocrisy and behavioral hypocrisy. Hypocrisy attribution is the judgment that an enterprise is hypocritical in essence; for example, stakeholders believe that an enterprise is hypocritical and two-faced (Wagner et al., 2020). Moral hypocrisy and behavioral hypocrisy lead to hypocrisy attribution, and moral hypocrisy is the leading factor of hypocrisy attribution. The perception of behavioral hypocrisy has a weaker effect on hypocrisy attribution than does that of moral hypocrisy (Wagner et al., 2020).

Studies on corporate hypocrisy have examined the formation and influencing factors of consumer perception of corporate hypocrisy caused by CSR activities. These studies have clearly defined corporate hypocrisy and have indicated that perception of corporate hypocrisy by consumers in response to CSR activities has a series of negative effects on enterprises. However, studies have not examined what measures should be adopted by enterprises to reduce the negative effect of consumers perceiving their CSR to be hypocritical. Expanding on relevant literature on corporate hypocrisy, we investigated what countermeasures should be adopted by enterprises to reduce the aforementioned negative effect and the influences of these countermeasures.

### Situational Crisis Communication Theory

This study is mainly based on SCCT, which is a crisis response theory proposed by Coombs (2007). According to SCCT, firms should select appropriate crisis response strategies by evaluating the nature of a crisis and the responsibility the firm bears for it (Coombs, 2007).

Attribution theory is a crucial theory of social psychology and a vital theoretical base of SCCT (Coombs, 2007). According to attribution theory, people’s attribution of responsibility to events affects their emotional reaction to these events, which can include anger or sympathy (Coombs, 2007). Moreover, people’s attribution of responsibility and their emotional reactions influence their subsequent behaviors (Coombs, 2007). Depending on an individual’s attribution of event responsibility, they would exhibit negative or positive behavioral reactions (Weiner, 2006). According to SCCT, the attribution of stakeholders has strong effects on their understanding of organizational crisis responsibility (Coombs and Holladay, 2001) and their emotional and behavioral responses toward an organization (Coombs and Holladay, 2005). If stakeholders believe that an organization is responsible for a crisis, they feel angry and engage in negative word of mouth toward the organization, which negatively affects the reputation of the organization (Coombs, 2007).

When a crisis event occurs, people conduct attribution analysis of the root causes of the event to assign responsibility for the event (Jeong, 2009). Whether enterprises can control the occurrence of a crisis is a crucial factor that influences consumers’ responsibility attribution and moral judgment (Weiner, 2006); the enterprise’s response to a crisis is determined by the company’s abilities and motivation (Malle and Knobe, 1997; Heider, 2013). According to SCCT, crises can be divided into three categories depending on the results of attribution: victim, accidental, and preventable crises (Coombs, 2007). A victim crisis is a situation in which an enterprise is considered the victim of a crisis, and the degree of responsibility assigned to enterprises for such a crisis is very low. An under accidental crisis is a situation in which a crisis is considered unintentional and enterprises are regarded as being unable to control the crisis. A moderate degree of responsibility is assigned to enterprises for such a crisis. A preventable crisis is also called an intentional crisis. Such crises are considered to be intentional, purposeful, and controllable, and high responsibility is assigned to enterprises for such a crisis (Coombs and Holladay, 2001). On the basis of the aforementioned information, moral hypocrisy corresponds to preventable crises, and behavioral hypocrisy corresponds to victim and accidental crises.

According to SCCT, CSR communication is a crucial crisis response strategy (Coombs, 2007). According to the order in which crisis information and CSR communication strategy information is presented, CSR communication strategies can be divided into proactive and reactive CSR communication strategies (Murray and Vogel, 1997; Chen et al., 2019). The effectiveness of CSR communication depends on consumers’ attribution and judgment of motivation. Whether the motivation of CSR communication is attributed to self-interest or altruism directly affects the influence of CSR communication (Becker-Olsen et al., 2006; Yoon et al., 2006). Therefore, an appropriate CSR communication strategy must be selected according to the relevant crisis (Coombs and Holladay, 2001).

We introduced SCCT into research on response strategies for hypocrisy crises and examined the effectiveness of different types of response strategies (reactive CSR communication vs. proactive CSR communication) under different types of hypocrisy crises (moral hypocrisy vs. behavioral hypocrisy) to determine the most effective response strategy under different types of hypocrisy crises.

### Negative Emotions

Psychological research has indicated that moral transgression leads to a negative emotional response from people and that the violation of moral standards is closely related to immoral behavior (Tangney et al., 2007). A study identified three types of negative emotions, namely contempt, anger, and disgust (Haidt, 2003). When an individual clearly expresses disapproval of the behavior of a moral violator, the aforementioned negative emotions usually occur simultaneously (Gutierrez and Giner-Sorolla, 2007).

Some studies have indicated that negative emotions are generated from people’s evaluations of the consequences of behaviors and events. When people believe that some behaviors and practices threaten their legitimate welfare, negative emotions emerge. Some negative emotions, such as disgust and anger, arise in situations where some events are considered to be controlled by others (Lerner et al., 2004). People experience negative emotions when facing negative events that others can control or avoid (Watson and Spence, 2007). In addition, perception of fairness (Watson and Spence, 2007), violation of norms or moral standards (Watson and Spence, 2007), and violation of human dignity (Grappi et al., 2013) lead to negative emotions.

Negative emotions have various effects. They influence factors such as evaluation judgment (Zheng and Wang, 2022), behavior preference (Khatoon and Rehman, 2021), risk assessment (Tindall et al., 2021), word-of-mouth intention (Xiao et al., 2018), complaint behavior (Hoang, 2020), and satisfaction of needs (Avilov, 2021). Negative information conveyed to a person has a stronger influence on the person when they are in a negative emotional state (Jin and Oh, 2021). According to emotion repair theory, negative emotions encourage consumers to adopt response strategies, such as seeking social support to mitigate their anger, to seek an improvement in their environment (Mahapatra and Mishra, 2021).

Negative emotions have been considered in the study of CSR. When an enterprise violates moral standards because of its misconduct, a series of specific “moral emotions” are generated (Lefebvre and Krettenauer, 2019). The negative emotions of consumers toward the CSR activities of enterprises are caused by the moral transgression behavior of enterprises (Grappi et al., 2013). If CSR activities do not meet the moral standards of consumers, they experience negative emotions (Su et al., 2014). Irresponsible corporate behavior can prompt negative emotions among consumers, which leads to consumers exhibiting adverse behavior toward enterprises (Xie et al., 2015). Consumers’ negative emotions also influence their response to corporate hypocrisy. Consumers’ internal attribution of CSR hypocrisy and perception of hypocrisy can lead to negative emotions among them (Wang et al., 2020; Wang and Zhu, 2020). The perception of moral hypocrisy and hypocrisy attribution is more likely than that of behavior hypocrisy to lead to negative emotional reactions, such as anger, contempt, and disgust, among stakeholders (Wagner et al., 2020).

This study introduced negative emotions into the investigation of responses to hypocrisy crises. By analyzing the distinct perceptions of consumers in response to different CSR communication strategies under various types of hypocrisy crises, we investigated the effects of these perceptions on consumers’ negative behavior and the role of negative emotions in these effects.

### Conceptual Model

Wagner et al. (2020) divided corporate hypocrisy into moral hypocrisy and behavioral hypocrisy. Moral hypocrisy originates from the selfish motivations of enterprises and is driven by deceptive behavior. By contrast, behavioral hypocrisy is caused by the unintentional behavior of enterprises, driven by the inconsistency between the words and deeds of enterprises, and not associated with moral problems (Wagner et al., 2020). The aforementioned division of corporate hypocrisy is consistent with the basic notion of SCCT. The perception of whether corporate hypocrisy is controllable influences people’s attribution of responsibility for the hypocrisy and evaluation of moral problems associated with the hypocrisy. Reasonable countermeasures in response to people’s perception of corporate hypocrisy can be developed only by obtaining a clear understanding of the nature of the relevant crisis. On the basis of the aforementioned analysis, we divided hypocrisy crises into moral and behavioral hypocrisy crises, which were considered as moderator variables in the research model.

According to SCCT, CSR communication is a crucial crisis communication strategy (Coombs, 2007). A suitable CSR communication strategy can help enterprises resolve corporate hypocrisy crisis, improve their operation, regain confidence in CSR investment, and increase their level of CSR investment. Consumers’ perception of the motivation of a CSR communication strategy influences the ultimate effects of the implementation of this strategy. On the basis of consumer perceptions, the CSR communication behavior of enterprises can be divided into self-interest-driven and altruism-driven behaviors (Becker-Olsen et al., 2006). Studies have indicated that a proactive CSR communication strategy is associated with altruistic qualities (altruistic motivation), whereas a reactive CSR communication strategy is often associated with self-interest (Groza et al., 2011). In specific situations, the effect of a reactive CSR communication strategy might be superior to that of a proactive CSR communication strategy. For example, in the evaluation of an enterprise, when the CSR information of the enterprise contradicts its moral performance in subsequent crisis events, that CSR communication strategy results in people perceiving the enterprise as hypocritical (Wagner et al., 2009; Rim, 2013). The CSR communication strategy most appropriate in a given situation is determined by the corresponding scenario. For example, under different product harm crisis situations, the same CSR communication strategy can have different effects on consumers’ attitudes toward enterprises (Chen et al., 2019). Similarly, in line with SCCT, the CSR communication strategy must be matched with the corresponding crisis situation to effectively resolve the crisis (Coombs and Holladay, 2001). On the basis of the aforementioned analysis, we divided response strategies to hypocrisy crises into reactive and proactive CSR communication strategies, which were considered as independent variables in the research model.

Consumers’ perception of corporate hypocrisy is often affected by the motivation attributed by them to corporate behavior. When consumers perceive dishonest motivation, they experience negative emotions (Wang and Wang, 2014), which directly lead to them exhibiting irrational behavior. When consumers perceive enterprises to be hypocritical, they feel cheated, which results in negative emotions, such as hatred, anger, and contempt. In addition, this perception reduces their devotion to the enterprise and results in them engaging in adverse actions against the enterprise, such as making negative comments, making product complaints, and engaging in product boycotts. In this study, negative emotions (contempt, anger, and disgust) were selected as mediator variables, and negative behaviors (negative word of mouth, complaint, and boycott) were selected as dependent variables.

**Figure 1:**
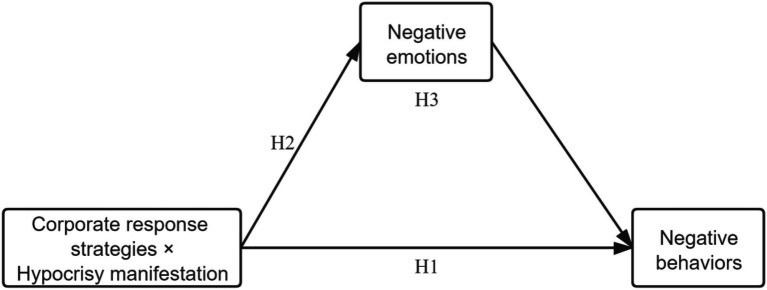
Conceptual model of this study.

The conceptual model of this study is displayed in Figure 1. This study explored the effect of the match between hypocrisy manifestation (moral hypocrisy vs. behavioral hypocrisy) and the corporate response strategy (reactive CSR communication vs. proactive CSR communication) on consumers’ negative behaviors and the mediating influence of negative emotions on this effect.

### Hypotheses

#### Effect of the Match Between Hypocrisy Manifestation and the Corporate Response Strategy on Consumers’ Negative Behaviors

Consumers’ perception of the appropriateness, motivation, and timing of CSR activities can affect their beliefs, attitudes, and purchase intentions (Becker-Olsen et al., 2006). Consumers engage in negative behaviors, including engaging in negative word of mouth, making complaints, and engaging in boycotts (Xie et al., 2015), toward enterprises that exhibit irresponsible behavior toward the environment. Corporate hypocrisy can also lead to negative consumer behaviors, such as serious resistance to enterprises (Xiao et al., 2013) and refusal to buy the products and services of enterprises (Guèvremont, 2019). If consumers perceive an enterprise to be hypocritical, they exhibit a negative reaction to the enterprise to express their dissatisfaction. Some studies have reported that high performance in CSR activities helps enterprises obtain favorable attribution from consumers and reduces enterprise condemnation by consumers (Klein and Dawar, 2004). However, consumers’ evaluation of CSR behavior depends on whether this behavior is considered sincere (Yoon et al., 2006). When consumers are exposed to information related to a corporate crisis and CSR communication strategy, their perception regarding whether the enterprise is self-interested or altruistic depends on their comprehensive evaluation of this information (Rim, 2013).

When a moral hypocrisy crisis occurs, the behavior of the enterprise is regarded as intentional, and the enterprise is trusted to have the ability to control the crisis. In this scenario, the enterprise bears considerable responsibility and faces moral condemnation. SCCT emphasizes that when an organization causes a crisis because of its misconduct, it should take remedial measures and actively commit to improvement for handling the crisis appropriately (Coombs, 2007). If the enterprise adopts a reactive CSR communication strategy in the aforementioned scenario, consumers would perceive the enterprise as sincerely attempting to correct its mistakes after engaging in immoral behavior; thus, the negative behaviors of consumers would decrease. By contrast, if the enterprise adopts a proactive CSR communication strategy in the aforementioned scenario, the crisis information would contradict the CSR information. Therefore, consumers would not change their attribution of corporate moral hypocrisy, and they would consider the think that CSR communication made before the crisis to be hypocritical deception. Consequently, the negative behaviors of consumers would not decrease. On the basis of the aforementioned information, this study infers that in the case of a moral hypocrisy crisis, adopting a reactive CSR communication strategy is a superior approach to adopting a proactive CSR communication strategy for responding to the crisis.

When a behavioral hypocrisy crisis occurs, the behavior of the enterprise is regarded as being unintentional, and the enterprise is considered to not have the ability to control the crisis. In this scenario, the enterprise is attributed low responsibility for the crisis and is not condemned at the moral level. However, a behavioral crisis still generates negative emotions in consumers, and the enterprise faces the negative effect caused by the hypocrisy crisis. In the aforementioned scenario, if the enterprise adopts a reactive CSR communication strategy, consumers would consider that the enterprise is taking temporary measures to please the public in response to the pressure resulting from the crisis (Murray and Vogel, 1997). Thus, consumers would have doubts about the motivation of the enterprise, and their negative behaviors would not considerably decrease. By contrast, if the enterprise adopts a proactive CSR communication strategy in the aforementioned scenario, the enterprise can earn goodwill that can compensate for the negative effects of its subsequent unintentional hypocritical behavior. In this case, the CSR communication would likely to be attributed to sincere public interest rather than external pressure (Chen et al., 2019). The resultant positive image created for the enterprise would increase consumers’ trust in it, and consumers would believe that the enterprise committed an unintentional error; thus, consumers’ negative behaviors would be considerably reduced. Consequently, this study infers that in the case of a behavioral hypocrisy crisis, adopting a proactive CSR communication strategy is a better option than is adopting a reactive CSR communication strategy for responding to the crisis.

In conclusion, the interaction between hypocrisy manifestation and the corporate response strategy can affect consumers’ negative behaviors. Therefore, the following hypotheses are proposed:

*H1*: The interaction between hypocrisy manifestation (moral hypocrisy vs. behavioral hypocrisy) and the corporate response strategy (reactive CSR communication vs. proactive CSR communication) would have a significant effect on consumers’ negative behaviors.

*H1a*: During a moral hypocrisy crisis, a reactive CSR communication strategy (compared with a proactive CSR communication strategy) would significantly reduce consumers’ negative behaviors.

*H1b*: During a behavioral hypocrisy crisis, a proactive CSR communication strategy (compared with a reactive CSR communication strategy) would significantly reduce consumers’ negative behaviors.

#### Effect of the Match Between Hypocrisy Manifestation and the Corporate Response Strategy on Consumers’ Negative Emotions

Negative consumer emotions refer to complex emotions caused by major inconsistencies in consumers’ emotional experience (Nawijn and Biran, 2019). Marketing and consumer behavior studies have indicated that emotional response has a crucial effect on consumers’ response to corporate social irresponsibility (CSI; Kang et al., 2016; Antonetti and Maklan, 2017). CSI can stimulate various complex negative emotions, such as anger (Xie et al., 2015), disgust (Xie et al., 2015), contempt (Romani et al., 2013), moral indignation (Lindenmeier et al., 2012), fear (Antonetti and Maklan, 2016), sadness (Antonetti and Maklan, 2016), and dissatisfaction (Romani et al., 2012). The stimulation of negative emotions results in consumers being eager to punish those who commit mistakes and to influence them to correct their inappropriate behaviors (Hofmann et al., 2018). Consumers’ perception of corporate hypocrisy (Wang et al., 2020; Wang and Zhu, 2020) and their internal attribution of corporate hypocrisy (Wang and Zhu, 2020) prompt a negative emotional response. Whether consumers attribute the motivation of a CSR communication strategy to public interest or self-interest determines whether this strategy can improve the negative emotions generated in consumers by a hypocrisy crisis.

When a moral hypocrisy crisis occurs, the behavior of the enterprise is regarded as a moral transgression with ulterior motives, which stimulates strong negative emotions in consumers. In this scenario, if the enterprise adopts a reactive CSR communication strategy, consumers would consider that the enterprise is attempting to correct its mistakes sincerely after engaging in immoral behavior; thus, the negative emotions of consumers would reduce. By contrast, if the enterprise adopts a proactive CSR communication strategy and then faces a moral hypocrisy crisis, the crisis information contradicts the CSR information. Therefore, consumers would not change their attribution of corporate moral hypocrisy, and they would consider the CSR communication made before the crisis to be hypocritical deception. Consequently, the negative emotions of consumers would not decrease.

A behavioral hypocrisy crisis stimulates less negative emotions in consumers than does a moral hypocrisy crisis; however, a behavioral hypocrisy crisis still generates negative emotions in consumers, and the enterprise faces the adverse impact caused by these emotions. In this scenario, if the enterprise adopts a reactive CSR communication strategy, consumers would consider that the enterprise is adopting temporary measures to please the public in response to external pressure, and these measures are likely to be attributed to self-interest. Therefore, the negative emotions of consumers would not decrease markedly. By contrast, if the enterprise adopts a proactive CSR communication strategy, the enterprise would earn the goodwill of consumers. In this case, consumers would be likely to attribute the motivation of CSR communication to public interest rather than external pressure. The positive image cultivated by the enterprise would increase consumers’ trust in the enterprise. Thus, consumers’ negative emotions would decrease significantly.

Thus, the interaction between hypocrisy manifestation and the corporate response strategy can affect consumers’ negative emotions. Therefore, the following hypotheses are proposed:

*H2*: The interaction between hypocrisy manifestation (moral hypocrisy vs. behavioral hypocrisy) and the corporate response strategy (reactive CSR communication vs. proactive CSR communication) would have a significant effect on consumers’ negative emotions.

*H2a*: During a moral hypocrisy crisis, a reactive CSR communication strategy (compared with a proactive CSR communication strategy) would significantly reduce consumers’ negative emotions.

*H2b*: During a behavioral hypocrisy crisis, a proactive CSR communication strategy (compared with a reactive CSR communication strategy) would significantly reduce consumers’ negative emotions.

#### Mediating Role of Negative Emotions

Emotion directly affects behavior because emotion can stimulate and drive behavior (Steinhauser et al., 2018). Individual emotional responses to events can lead to corresponding behavioral responses (Steinhauser et al., 2018). Studies have indicated that consumers consider irresponsible behavior on environmental issues by enterprises as a moral transgression, which stimulates negative emotions in consumers. These emotions then trigger negative behaviors, including making negative comments, making complaints, and engaging in boycotts (Xie et al., 2015). When investigating CSI, researchers have often used emotion to explain consumer behavior (Antonetti, 2020) and have considered negative emotions as mediating variables to explain consumers’ negative behaviors (Lindenmeier et al., 2012). Studies have confirmed the role of negative emotions in the perception of corporate hypocrisy. The perception of corporate hypocrisy indirectly affects consumers’ negative behaviors by affecting consumers’ negative emotions (Wang et al., 2020; Wang and Zhu, 2020). Therefore, firms should respond to a hypocrisy crisis by reducing consumers’ negative behaviors by mitigating their negative emotions.

Specifically, when a moral hypocrisy crisis occurs, if the enterprise adopts a reactive CSR communication strategy, consumers would consider that the enterprise is attempting to correct its fault; however, if the enterprise adopts a proactive CSR communication strategy, the perception of corporate moral hypocrisy by consumers would be deepened, and consumers would have a strong perception that the corporate behavior is driven by self-interest. Therefore, under the perception of moral hypocrisy, a reactive CSR communication strategy would substantially reduce consumers’ negative emotions and negative behaviors. By contrast, when a behavioral hypocrisy crisis occurs, if the enterprise adopts a reactive CSR communication strategy, consumers would consider that the enterprise is taking measures to alleviate the external pressure caused by the hypocrisy crisis, and they would consider the CSR communication behavior to be driven by self-interest. However, if the enterprise adopts a proactive CSR communication strategy when a behavioral hypocrisy crisis occurs, consumers would consider the CSR communication to be driven by public interest. Therefore, under the perception of behavioral hypocrisy, a proactive CSR communication strategy reduces consumers’ negative emotions and indirectly reduces their negative behaviors.

Thus, consumers’ negative emotions mediate the effect of the interaction between hypocrisy manifestation and the corporate response strategy on consumers’ negative behaviors. Therefore, the following hypothesis is proposed:

*H3*: Negative emotions would mediate the effect of the interaction between hypocrisy manifestation (moral hypocrisy vs. behavioral hypocrisy) and the corporate response strategy (reactive CSR communication vs. proactive CSR communication) on consumers’ negative behaviors.

## Research Design

A situational simulation experiment was conducted in this study. The data obtained from this experiment were analyzed using AMOS 24.0 and SPSS 22.0. We designed a questionnaire describing 12 situations (2 × 3 × 2) related to corporate behavior to measure consumers’ perceptions toward hypocrisy manifestation type, negative emotion, and negative behavior. Confirmatory factor analysis was used to test the reliability and validity of the measurements. Independent-sample *t*-test and paired-sample *t*-test were used to measure the effectiveness of the manipulation. Moreover, two-way analysis of variance and independent-sample *t*-test were used to test consumers’ reactions to different corporate response strategies.

### Experiment Design

This study examined the effect of different response strategies on consumers’ negative behaviors under various hypocrisy situations and the mediating influence of consumers’ negative emotions on this effect. Therefore, a 2 (hypocrisy manifestation: moral hypocrisy vs. behavioral hypocrisy) × 3 (corporate response strategy: reactive CSR communication vs. proactive CSR communication vs. no strategy) × 2 (scenario: food safety scenario vs. environmental protection scenario) between-group experimental design was adopted in this study.

### Stimulus Design

To enhance the external validity of the experiment and ensure that the research results were unaffected by the scenario type, we selected one case each focusing on “food safety” and “environmental protection” and named the investigated enterprise “enterprise A.” Two types of experimental stimuli were used in this study: the type of hypocrisy manifestation (moral hypocrisy or behavioral hypocrisy) and the corporate response strategy (a reactive CSR communication strategy, a proactive CSR communication strategy, and no strategy).

The moral hypocrisy incident described in the questionnaire is caused by the intentional immoral behavior of enterprise A, whereas the behavioral hypocrisy incident described in the questionnaire is caused by an unintentional inconsistency between the words and deeds of enterprise A. The description of the “food safety” scenario is as follows: enterprise A has long claimed to prioritize food hygiene and safety, has advocated for the production of healthy food, and has stated that it would donate 10 million yuan for the production and monitoring of safe food in poor areas with substandard health and medical conditions. The moral hypocrisy is that enterprise A produces food by using low-cost waste oil that does not meet hygiene standards. The behavioral hypocrisy is that enterprise A has only donated 500,000 yuan to poor areas because the departments receiving public welfare funds in these areas are corrupt. The description of the “environmental protection” scenario is as follows: enterprise A claims that it has been implementing the “million tree planting plan” for many years, attaches importance to environmental protection, and sows tens of thousands of saplings in areas experiencing desertification every year to address desertification in China. The moral hypocrisy is that the products of enterprise A are produced using low-cost packaging materials that do not meet environmental protection standards. The behavioral hypocrisy is that the tree planting plan of enterprise A was ceased 3 years earlier because the local government announced a unified plan for the transformation of areas experiencing desertification.

The reactive and proactive CSR communication strategies, respectively, described in the questionnaire is as follows: (1) enterprise A issues a CSR statement 2 months after the hypocrisy event is exposed and (2) enterprise A issues a CSR statement 2 months before the hypocrisy event is exposed. Finally, the no strategy situation described in the questionnaire is as follows: enterprise A does not issue a CSR statement, nor does it take any other actions. The CSR communication strategy information in the “food safety” case is as follows: enterprise A announced that it would donate 10% of its annual profits to the Chinese health and medical sector. The CSR communication strategy information in the “environmental protection” case is as follows: enterprise A announced that it would donate 10 million yuan to the Chinese environmental protection sector.

### Experimental Method and Procedure

In this study, the effectiveness of the manipulation of experimental materials was examined through a pretest. Both the pretest and formal experiment adopted the same scale. With regard to the hypocrisy manifestation, participants had to determine whether the corporate hypocrisy was moral hypocrisy or behavioral hypocrisy. A total of 40 individuals were randomly invited to participate in the pretest. These individuals were randomly assigned to different experimental situations for data collection.

During the formal experiment, we randomly distributed questionnaires on 12 experimental situations to individuals [2 (hypocrisy manifestation: moral hypocrisy vs. behavioral hypocrisy) × 3 (corporate response strategies: reactive CSR communication vs. proactive CSR communication vs. no strategy) × 2 (food safety scenario vs. environmental protection scenario)]. First, the purpose of this study was explained to the participants to dispel their concerns. The participants were then asked to read a paragraph of background materials, including an introduction to the considered enterprise, the CSR activities of the enterprise, the hypocrisy behavior of the enterprise, and the CSR communication strategy of the enterprise. The content related to corporate hypocrisy events was adapted from real cases to maximize credibility, and was written with a focus on readability. After reading the background materials, the participants were asked to answer questionnaire items related to moral hypocrisy and behavioral hypocrisy (the corresponding scores were used in for the manipulation test) as well as negative emotions and negative behaviors (the corresponding scores were used in the hypothesis test). Finally, the participants provided demographic information. After the participants completed the questionnaire, we thanked them and gave them small gifts.

### Variable Measurement

#### Hypocrisy Manifestation

The scales used in this study for measuring hypocrisy manifestation were developed through two-way translation and were based on the research of Wagner et al. (2009, 2020) and Alicke et al. (2013). The scale used for measuring moral hypocrisy contains three items: “Enterprise A pretends that it is not intentional…”; “Enterprise A deliberately deceives others through…”; and “Enterprise A tries to be more socially responsible than it actually is.” The scale used for measuring behavioral hypocrisy also contains three items: “What enterprise A says and does is different”; “Enterprise A’s words and deeds are inconsistent”; and “Enterprise A’s behavior is inconsistent with its external publicity.” All the aforementioned items were measured on a seven-point Likert scale ranging from 1 for “completely disagree” to 7 for “completely agree.”

#### Negative Emotions

The scale used in this study for measuring negative emotions was developed through two-way translation and was based on the study of Xie et al. (2015). This scale contains nine items: three items each related to contempt, anger, and disgust. The items related to contempt are as follows: “I scorn the behavior of enterprise A”; “I disdain the behavior of enterprise A”; and “I despise the behavior of enterprise A.” The items related to anger are as follows: “I am unhappy with the behavior of enterprise A”; “I am indignant at the behavior of enterprise A”; and “I am furious with the behavior of enterprise A.” The items related to disgust are as follows: “I am disgusted with the behavior of enterprise A”; “I dislike the behavior of enterprise A”; and “I detest the behavior of enterprise A.” All the aforementioned items were measured on the seven-point Likert scale.

#### Negative Behaviors

The scale used in this study for measuring negative behaviors was developed through two-way translation and was based on the study of Xie et al. (2015). This scale contains nine items: three, four, and two items related to negative word of mouth, complaints, and boycotts, respectively. The items related to negative word of mouth are as follows: “I will tell my relatives, friends, and others that enterprise A is not good”; “I will advise my relatives, friends, and others not to work for enterprise A”; and “I will tell my relatives, friends, and others that enterprise A has done a lot of bad things.” The items related to complaints are as follows: “I will complain directly to enterprise A”; “I will complain to the news media”; “I will complain to the government or industry authorities”; and “I will complain to the personnel of enterprise A.” The items related to boycott are as follows: “I will tell other enterprises not to do business with enterprise A” and “I will tell my friends not to buy the products of enterprise A.” All the aforementioned items were measured using the seven-point Likert scale.

## Data Analysis

### Sample Description

In this study, we conducted a random survey related to 12 experimental situations. To avoid the drawbacks of a pure student sample, we collected 420 valid questionnaires from college students and other social groups. The descriptive statistics of the respondents are presented in Table 1.

**Table 1:** Characteristics of the survey respondents.

Classification indicator	Category	Frequency	Percentage (%)
Gender	Male	175	41.7
	Female	245	58.3
Age	10–17	6	1.4
	18–25	338	80.5
	26–33	62	14.8
	34–41	7	1.7
	>42	7	1.7
Marital status	Married	32	7.6
	Single	388	92.4
Religious	Yes	19	4.5
	No	401	95.5
Occupation	Worker	6	1.4
	Farmer	3	0.7
	Government or institution personnel	51	12.1
	Student	269	64.0
	Individual operator	7	1.7
	Enterprise staff	55	13.1
	Technician	8	1.9
	Freelancer	21	5.0
Monthly income	<500	38	9.0
	500–1,000	93	22.1
	1,000–2,000	83	19.8
	2,000–3,000	61	14.5
	3,000–5,000	67	16.0
	5,000–8,000	54	12.9
	8,000–10,000	10	2.4
	>10,000	14	3.3
Education	Junior college or undergraduate	266	63.3
	Postgraduate or higher	154	36.7

### Reliability and Validity Test

#### Reliability Test

A reliability test was conducted to examine the reliability of the research data. The corrected item–total correlation was used to screen the measurement items, and the Cronbach’s *α* coefficient was used to test the internal consistency reliability of the adopted measurement scales.

The corrected item–total correlation of each measurement item was higher than 0.5, and the deletion of any item did not significantly improve the estimated Cronbach’s *α* coefficient; thus, all the measurement items were retained. Moreover, the estimated Cronbach’s *α* coefficients of all the items were higher than 0.8, which indicated that the adopted scales had favorable internal consistency reliability and that the research data had high reliability. The results of reliability analysis are presented in Table 2.

**Table 2:** Results of reliability analysis.

Latent variable		Item	CITC	CAID	Cronbach’s *α*
Hypocrisy manifestation	Moral hypocrisy	A1	0.644	0.861	0.876
		A2	0.705	0.851	
		A3	0.631	0.862	
	Behavioral hypocrisy	B1	0.729	0.846	
		B2	0.744	0.844	
		B3	0.630	0.863	
Negative emotions	Contempt	C1	0.835	0.971	0.972
		C2	0.851	0.970	
		C3	0.876	0.969	
	Anger	D1	0.885	0.969	
		D2	0.894	0.968	
		D3	0.877	0.969	
	Disgust	E1	0.878	0.969	
		E2	0.902	0.968	
		E3	0.907	0.968	
Negative behaviors	Negative word-of-mouth	F1	0.745	0.929	0.936
		F2	0.766	0.928	
		F3	0.782	0.927	
	Complaint	G1	0.722	0.931	
		G2	0.779	0.927	
		G3	0.750	0.929	
		G4	0.735	0.930	
	Boycott	H1	0.786	0.927	
		H2	0.763	0.928	

CITC, corrected item–total correlation and CAID, Cronbach’s alpha if item deleted.

#### Validity Test

The content validity and construct validity of the adopted scales were examined.

##### Content Validity

Content validity reflects the appropriateness and consistency of the questionnaire content. The items used to measure all the variables in this study were derived from the literature, extensively discussed with relevant experts, and appropriately modified according to the research background and research content. Therefore, the questionnaire measurement scale of this study had favorable content validity.

##### Construct Validity

Two types of construct validity exist: convergent validity and discriminant validity. Confirmatory factor analysis was performed using AMOS 24.0. The obtained fitting indices (*χ*^2^ = 290.901, *χ*^2^/df = 1.469, RMSEA = 0.033, GFI = 0.947, AGFI = 0.920, NFI = 0.972, RFI = 0.961, IFI = 0.991, TLI = 0.987, and CFI = 0.991) indicated that the measurement model had a high degree of fit with the research results.

According to the literature, three criteria must be met to achieve convergent validity. First, the normalized factor loading of all the measured items should be greater than 0.5 and reach the significance level. Second, the average variance extracted (AVE) should be higher than 0.5. Third, the composite reliability should be higher than 0.7. As presented in Table 3, all the test items fulfilled the aforementioned criteria. Thus, the adopted scales had favorable convergent validity.

**Table 3:** Normalized factor loading, average variance extracted (AVE), and composite reliability.

Latent variable		Item	Load	AVE	CR
Hypocrisy manifestation	Moral hypocrisy	A1	0.504	0.5100	0.8567
		A2	0.615		
		A3	0.559		
	Behavioral hypocrisy	B1	0.836		
		B2	0.904		
		B3	0.774		
Negative emotions	Contempt	C1	0.857	0.7790	0.9694
		C2	0.866		
		C3	0.907		
	Anger	D1	0.876		
		D2	0.882		
		D3	0.872		
	Disgust	E1	0.879		
		E2	0.894		
		E3	0.909		
Negative behaviors	Negative word-of-mouth	F1	0.837	0.5524	0.9158
		F2	0.892		
		F3	0.831		
	Complaint	G1	0.592		
		G2	0.681		
		G3	0.663		
		G4	0.575		
	Boycott	H1	0.732		
		H2	0.815		

AVE, average variance extracted and CR, composite reliability.

If the square root of the AVE of each latent variable is higher than the correlation coefficient between the variable and the other variables, the measurement scale has favorable discriminant validity. As presented in Table 4, all the adopted scales met the aforementioned conditions. Therefore, these scales had favorable discriminant validity.

**Table 4:** Square root of the AVE and correlation coefficient matrix.

	Hypocrisy manifestation	Negative emotions	Negative behaviors
Hypocrisy manifestation	(0.714)		
Negative emotions	0.623	(0.883)	
Negative behaviors	0.455	0.647	(0.743)

The diagonal data of the matrix represent the square roots of the AVE values, and the lower half of the matrix represents the correlation coefficients.

#### Manipulation Test

In the formal experiment, a manipulation test was conducted to test whether the participants could accurately distinguish the types of corporate hypocrisy to ensure that moral hypocrisy and behavioral hypocrisy were effectively manipulated.

The participants assigned to the moral hypocrisy and behavioral hypocrisy groups exhibited significant differences in their perceptions and judgment of whether the enterprise causing the hypocrisy event had moral problems (M_moral hypocrisy_ = 5.1320 > M_behavioral hypocrisy_ = 4.0595; *t* = 8.885, *p* < 0.01). The aforementioned result indicates that the experiment was successful in manipulating whether the enterprise had moral problems; in addition, the participants in the moral hypocrisy and behavioral hypocrisy groups exhibited significant differences in their perception and judgment of whether inconsistency existed between the words and deeds of the enterprise (M_moral hypocrisy_ = 5.7150 > M_behavioral hypocrisy_ = 4.9484; *t* = 6.026, *p* < 0.01). The aforementioned result indicates that the participants experienced a stronger perception of inconsistency between the words and deeds of the enterprise when they perceived the enterprise as having moral problems than when they did not have this perception.

The aforementioned finding was verified by the following results. In the moral hypocrisy group, the participants’ perception and judgment of the existence of moral problems and the inconsistency between the words and deeds of the enterprise were significantly different (M_morality (moral hypocrisy group)_ = 5.1320 < M_behavior (moral hypocrisy group)_ = 5.7150; *t* = −8.380, *p* < 0.01). Similar results were obtained for the behavioral hypocrisy group (M_morality (behavioral hypocrisy group)_ = 4.0595 < M_behavior (behavioral hypocrisy group)_ = 4.9484; *t* = −9.155, *p* < 0.01).

In summary, corporate hypocrisy was successfully manipulated in the experiment.

#### Hypothesis Testing

##### Negative Behaviors

The statistical results indicated that compared with the behavioral hypocrisy crisis, the moral hypocrisy crisis produced higher scores for negative behaviors (i.e., more negative behaviors; M_moral hypocrisy_ = 4.1911 > M_behavioral hypocrisy_ = 3.3229; *t* = 7.338, *p* < 0.01).

The effect of the interaction between hypocrisy manifestation and the corporate response strategy on the participants’ negative behaviors was significant (*F* = 13.706, *p* < 0.01); the main effect of hypocrisy manifestation on the participants’ negative behaviors was significant (*F* = 41.233, *p* < 0.01); and the corporate response strategy had no significant main effect on the participants’ negative behaviors (*F* = 0.742, *p* > 0.05; Table 5).

**Table 5:** Results of the intersubject effect test.

Source	Square sum of type III	Degree of freedom	Mean square	*F*	Sig.
Hypocrisy manifestation	53.310	1	53.310	41.233	0.000
Corporate response strategies	0.959	1	0.959	0.742	0.390
Hypocrisy manifestation*Corporate response strategies	17.721	1	17.721	13.706	0.000
Error	354.256	274	1.293		
Total after modification	425.821	277			

Dependent variable: mean value of negative behaviors.

Under the moral hypocrisy crisis, different response strategies had significantly different effects on the participants’ scores for negative behaviors. Lower scores for negative behaviors (i.e., less negative behaviors) were observed for the adoption of the reactive CSR communication strategy than for the adoption of the proactive CSR communication strategy (M_reactive CSR communication_ = 3.9171 < M_proactive CSR communication_ = 4.3047; *t* = −2.130, *p* < 0.05). Compared with the adoption of no CSR communication strategy, the adoption of the reactive CSR communication strategy significantly reduced the participants’ scores for negative behaviors (M_reactive CSR communication_ = 3.9171 < M_no strategy_ = 4.3619; *t* = −2.259, *p* < 0.05). No significant difference was observed in the influences of the proactive CSR communication strategy and no CSR communication strategy on the participants’ scores for negative behaviors (M_proactive CSR communication_ = 4.3047, M_no strategy_ = 4.3619; *t* = −0.302, *p* > 0.05).

Under the behavioral hypocrisy crisis, different response strategies had significantly different effects on the participants’ scores for negative behaviors. Lower scores for negative behaviors were observed for the adoption of a proactive CSR communication strategy than for the adoption of a reactive CSR communication strategy (M_proactive CSR communication_ = 2.9233 < M_reactive CSR communication_ = 3.5460; *t* = 3.072, *p* < 0.01). Compared with the adoption of no CSR communication strategy, the adoption of the proactive CSR communication strategy significantly reduced the participants’ scores for negative behaviors (M_proactive CSR communication_ = 2.9233 < M_no strategy_ = 3.5000; *t* = −2.572, *p* < 0.05). No significant difference was noted in the influences of the reactive CSR communication strategy and no CSR communication strategy on the participants’ negative behaviors (M_reactive CSR communication_ = 3.5460, M_no strategy_ = 3.5000; *t* = 0.218, *p* > 0.05).

**Figure 2:**
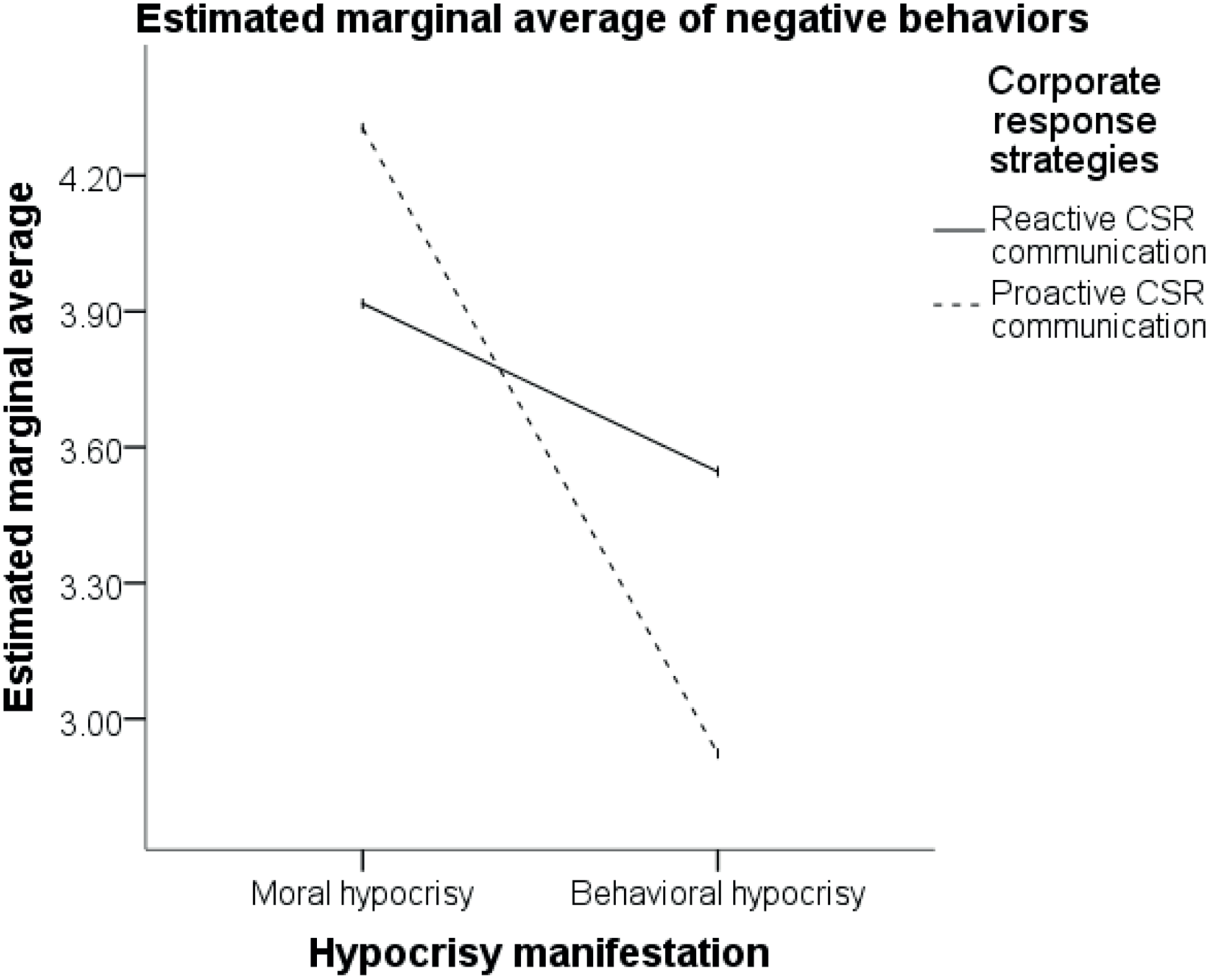
Interaction description.

**Figure 3:**
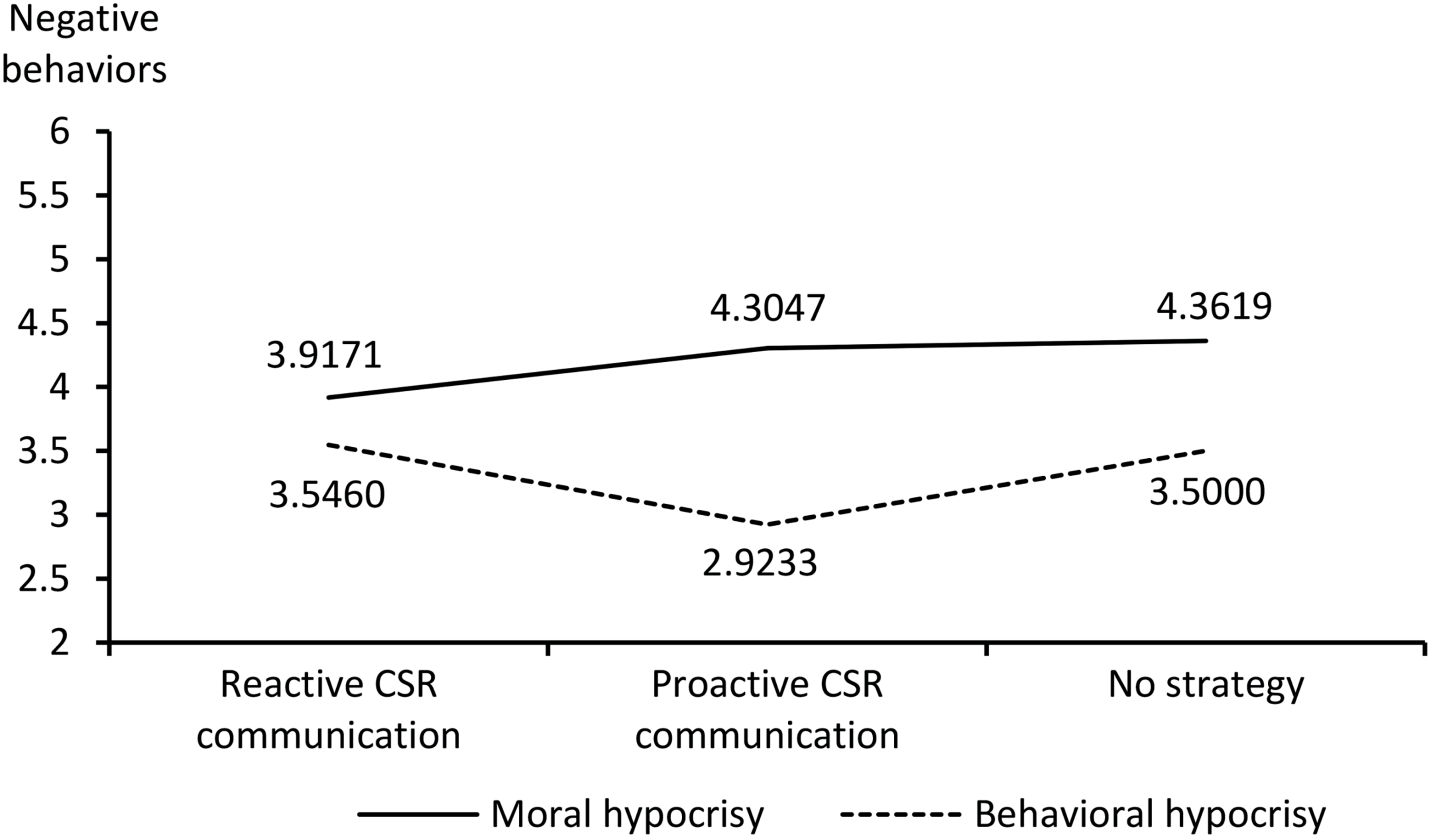
Trend of negative behaviors.

The aforementioned results (Figures 2, 3) support hypotheses H1, H1a, and H1b.

##### Negative Emotions

The participants exhibited higher scores for negative emotions (i.e., more negative emotions) for the moral hypocrisy crisis than for the behavioral hypocrisy crisis (M_moral hypocrisy_ = 5.2700 > M_behavioral hypocrisy_ = 3.9468; *t* = 10.593, *p* < 0.01).

The effect of the interaction between hypocrisy manifestation and the corporate response strategy on the participants’ negative emotions was significant (*F* = 11.403, *p* < 0.01); the main effect of hypocrisy manifestation on the participants’ negative emotions was significant (*F* = 78.034, *p* < 0.01); and the corporate response strategy had no significant main effect on the participants’ negative emotions (*F* = 0.000, *p* > 0.05; Table 6).

**Table 6:** Results of the intersubject effect test.

Source	Square sum of type III	Degree of freedom	Mean square	*F*	Sig.
Hypocrisy manifestation	127.199	1	127.199	78.034	0.000
Corporate response strategies	0.001	1	0.001	0.000	0.983
Hypocrisy manifestation*Corporate response strategies	18.587	1	18.587	11.403	0.001
Error	446.634	274	1.630		
Total after modification	591.104	277			

Dependent variable: mean value of negative emotions.

Under the moral hypocrisy crisis, different response strategies had significantly different effects on the participants’ negative emotions. Lower scores for negative emotions (i.e., less negative emotions) were observed for the adoption of the reactive CSR communication strategy than for the adoption of the proactive CSR communication strategy (M_reactive CSR communication_ = 4.9186 < M_proactive CSR communication_ = 5.4327; *t* = −2.530, *p* < 0.05). Compared with the adoption of no CSR communication strategy, the adoption of the reactive CSR communication strategy yielded significantly lower scores for negative emotions (M_reactive CSR communication_ = 4.9186 < M_no strategy_ = 5.4730; *t* = −2.757, *p* < 0.01). No significant difference was observed in the influences of the proactive CSR communication strategy and no CSR communication strategy on the participants’ scores for negative emotions (M_proactive CSR communication_ = 5.4327, M_no strategy_ = 5.4730; *t* = −0.208, *p* > 0.05).

Under the behavioral hypocrisy crisis, different response strategies had significantly different effects on the participants’ scores for negative emotions. Lower scores for negative emotions were observed for the adoption of a proactive CSR communication strategy than for the adoption of a reactive CSR communication strategy (M_proactive CSR communication_ = 3.5618 < M_reactive CSR communication_ = 4.0825; *t* = 2.278, *p* < 0.05). Compared with the adoption of no CSR communication strategy, the adoption of the proactive CSR communication strategy yielded significantly lower participant scores for negative emotions (M_proactive CSR communication_ = 3.5618 < M_no strategy_ = 4.1944; *t* = −2.787, *p* < 0.01). No significant difference was noted in the influences of the reactive CSR communication strategy and no CSR communication strategy on the participants’ negative emotions (M_reactive CSR communication_ = 4.0825, M_no strategy_ = 4.1944; *t* = −0.515, *p* > 0.05).

**Figure 4:**
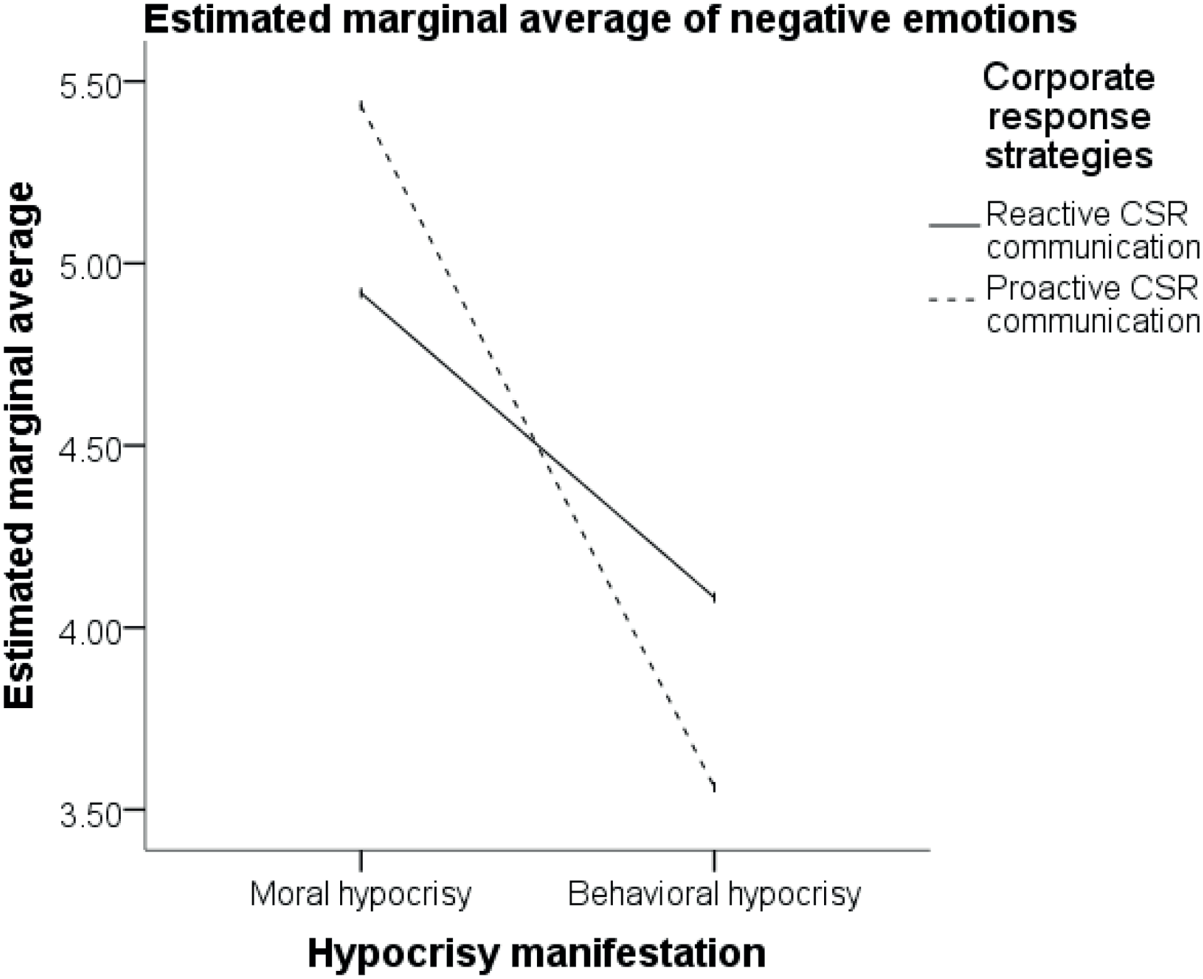
Interaction description.

**Figure 5:**
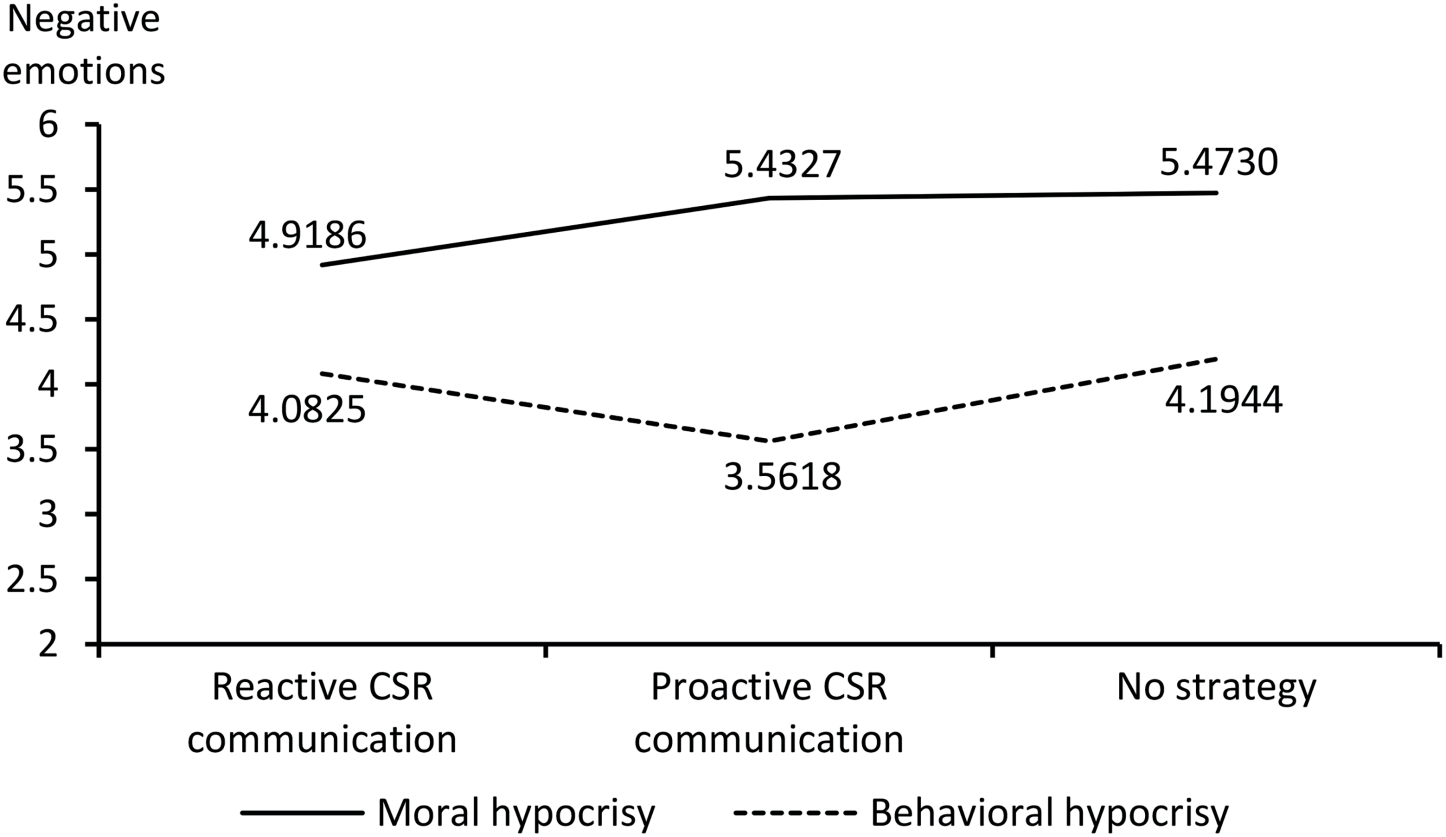
Trend of negative emotions.

The aforementioned results (Figures 4, 5) support hypotheses H2, H2a, and H2b.

#### Mediating Effect Testing

The bootstrap method was used to test the adjusted mediating effect of negative emotions on the effect of the interaction between hypocrisy manifestation and the corporate response strategy on negative behaviors (Preacher and Hayes, 2004; Chen et al., 2013). The independent variable in this test was the corporate response strategy (the reactive and proactive CSR communication strategies were coded as 1 and 2, respectively); the moderator variable was hypocrisy manifestation (the moral hypocrisy was coded as 1, and the behavioral hypocrisy was coded as 2); the mediator variable was negative emotions; and the dependent variable was negative behaviors. In bootstrap sampling, the nonparametric percentile method was used for bias correction, the number of bootstrap samples was set as 5,000, and the confidence level for the CIs was set as 95%.

The results of bootstrap sampling indicated that the mediating effect of negative emotions had a value of −0.6302 and was significant (95% CI: lower-limit CI = −1.0108, upper-limit CI = −0.2605, excluding 0).

Under the moral hypocrisy crisis, the indirect effect had a value of 0.3130, with the CI being [0.0716, 0.5614] and excluding 0, thus confirming the indirect effect. The direct effect had a value of 0.0746, with the CI being [−0.2080, 0.3572] and including 0, thus confirming the direct effect. The aforementioned results indicate that consumers’ negative emotions completely mediate the effect of the interaction between hypocrisy manifestation and the corporate response strategy on consumers’ negative behavior during a moral hypocrisy crisis.

Under the behavioral hypocrisy crisis, the indirect effect had a value of −0.3171, with the CI being [−0.5918, −0.0422] and excluding 0, thus confirming the indirect effect. The direct effect had a value of −0.3056, with the CI being [−0.5842, −0.0270] and excluding 0, thus confirming the direct effect. The aforementioned results indicate that consumers’ negative emotions partially mediate the effect of the interaction between hypocrisy manifestation and the corporate response strategy on consumers’ negative behavior during a behavioral hypocrisy crisis.

**Figure 6:**
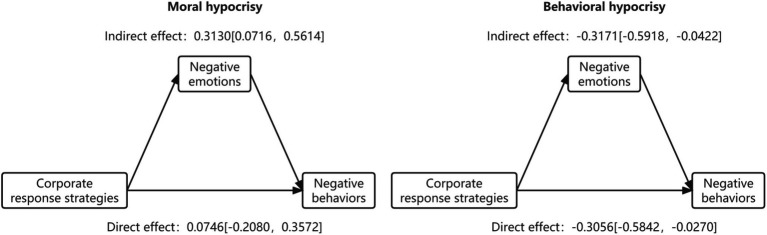
Description of mediating effect.

The results of bootstrap sampling support hypothesis H3. The descriptive results obtained for the mediating effect are presented in Figure 6.

## Discussion and Conclusion

### Discussion

This study explored the effect of the match between hypocrisy manifestation (moral hypocrisy vs. behavioral hypocrisy) and the adopted corporate response strategy (reactive CSR communication vs. proactive CSR communication) on consumers’ negative behaviors under a hypocrisy crisis. The results of this study indicate that consumers’ negative emotions mediate the aforementioned effect.

The present results reveal that the match between hypocrisy manifestation (moral hypocrisy vs. behavioral hypocrisy) and the adopted corporate response strategy (reactive CSR communication vs. proactive CSR communication) has a significant effect on consumers’ negative behaviors. Under different hypocrisy crisis situations, different CSR communication strategies must be adopted to reduce consumers’ negative behaviors appropriately. The aforementioned findings are in line with SCCT, which indicates that the adopted crisis communication strategy must match the crisis situation to ensure that the crisis is suitably resolved (Coombs and Holladay, 2001). In contrast to SCCT, which focuses on organizational reputation (Coombs, 2007), we examined the consumer response during a hypocrisy crisis, mainly focusing on the negative behaviors of consumers against enterprises.

In this study, corporate hypocrisy crises were divided into moral and behavioral hypocrisy crises. SCCT is based on the notion of attribution. It emphasizes that the nature of a crisis should be understood before a crisis communication strategy is selected. The nature of a hypocrisy crisis mainly depends on whether consumers consider an enterprise to be intentionally or unintentionally responsible for the crisis and whether the enterprise acts according to moral standards (Coombs and Holladay, 2001; Coombs, 2007). Therefore, the division of corporate hypocrisy crises in this study is consistent with SCCT. However, on the basis of SCCT, crises are divided into three categories: victim, accidental, and preventable crises (Coombs, 2007). Enterprises bear different degrees of responsibility for these three types of crises. The crisis categories based on SCCT and the crisis categories of this study have different forms. However, considering the nature of the crises, the two crisis categories of this study can correspond to the three crises based on SCCT.

The results of this study indicate that enterprises should consider consumers’ attribution of the motivation of firms’ response strategies when handling a hypocrisy crisis. Studies have indicated that the effectiveness of CSR communication depends on consumers’ attribution and judgment regarding firms’ motivation. Whether consumers perceive the motivation of CSR communication to be selfish or altruistic directly influences the effect of CSR communication (Becker-Olsen et al., 2006; Yoon et al., 2006; Chen et al., 2019), which in turn affects corporate reputation and consumers’ attitude and behavior toward enterprises. The aforementioned finding of this study is consistent with the results in the literature. In contrast to previous studies, we investigated consumers’ attribution of the motivation of corporate response strategies during a hypocrisy crisis.

The results of this study reveal that enterprises can reduce consumers’ negative behaviors during a hypocrisy crisis by reducing consumers’ negative emotions. Research on corporate hypocrisy has indicated that consumers’ negative emotions mediate the effect of hypocrisy perception on consumers’ behavioral response (Wang et al., 2020; Wang and Zhu, 2020). The results of this study are consistent with those of the aforementioned research. This study also found that consumers’ negative emotions exert a mediating influence on the effect of response strategies for a corporate hypocrisy crisis on consumer behavior. Thus, during a hypocrisy crisis, enterprises can adopt suitable response strategies to reduce consumers’ negative emotions for mitigating their negative behaviors.

This study examined the response strategies for corporate hypocrisy crises. Studies on corporate crisis management have suggested that enterprises should adopt different response strategies in according to different corporate crisis situations. For example, research on brand crisis management has indicated that strategies of denial, rational interpretation, or behavior correction should be adopted according to the attributes of a brand crisis (Dutta and Pullig, 2011). During a flight delay crisis, airlines should select an appropriate apology mode (responsibility orientation vs. sympathy orientation) that matches the delay attribution (internal attribution vs. external attribution; Chung and Lee, 2021). In line with previous relevant studies, we found that an appropriate corporate response strategy should be selected according to the type of corporate crisis to improve the effect of the implemented strategy. This study examined the crisis response strategies of enterprises during a corporate hypocrisy crisis and thus enriches research on corporate crisis response.

### Conclusion

This study examined the effects of different corporate response strategies on consumers’ negative behaviors under different hypocrisy crises. The following conclusions were obtained from the results of this study:

1. The interaction between hypocrisy manifestation (moral hypocrisy vs. behavioral hypocrisy) and the adopted corporate response strategy (reactive CSR communication vs. proactive CSR communication) significantly affects consumers’ negative behaviors. When a moral hypocrisy crisis occurs, different response strategies have significantly different effects on consumers’ negative behaviors. Under a moral hypocrisy, enterprises can reduce consumers’ negative behaviors by a significantly greater extent when adopting a reactive CSR communication strategy than when adopting a proactive CSR communication strategy. Similarly, when a behavioral hypocrisy crisis occurs, different response strategies have significantly different effects on consumers’ negative behaviors. Under a behavioral hypocrisy crisis, enterprises can reduce consumers’ negative behaviors by a significantly greater extent when adopting a proactive CSR communication strategy than when adopting a reactive CSR communication strategy.
2. The interaction between hypocrisy manifestation (moral hypocrisy vs. behavioral hypocrisy) and the corporate response strategy (reactive CSR communication vs. proactive CSR communication) significantly affects consumers’ negative emotions. When a moral hypocrisy crisis occurs, different response strategies have significantly different effects on consumers’ negative emotions. Under a moral hypocrisy, enterprises can reduce consumers’ negative emotions by a significantly greater extent when adopting a reactive CSR communication strategy than when adopting a proactive CSR communication strategy. Similarly, when a behavioral hypocrisy crisis occurs, different response strategies have significantly different effects on consumers’ negative emotions. Under a behavioral hypocrisy crisis, enterprises can reduce consumers’ negative emotions by a significantly greater extent when adopting a proactive CSR communication strategy than when adopting a reactive CSR communication strategy.
3. Negative emotions mediate the effect of the interaction between hypocrisy manifestation (moral hypocrisy vs. behavioral hypocrisy) and the corporate response strategy (reactive CSR communication vs. proactive CSR communication) on consumers’ negative behaviors. When a moral or behavioral hypocrisy crisis occurs, the CSR communication strategies adopted by enterprises indirectly affect consumers’ negative behaviors by affecting their negative emotions.
4. Different types of corporate hypocrisy crises (moral hypocrisy vs. behavioral hypocrisy) have significantly different effects on consumers’ negative emotions and negative behaviors. Specifically, a moral hypocrisy crisis produces more negative customer emotions and behaviors than does a behavioral hypocrisy crisis.

### Theoretical Contributions

The results of this study, which are in line with SCCT and previous research on corporate hypocrisy, indicate that enterprises should adopt a suitable CSR communication strategy according to the nature of the hypocrisy crisis. The theoretical contributions of this study are as follows:

1. This study enriches the literature on corporate hypocrisy. Studies have examined the definition of corporate hypocrisy, the effect of corporate hypocrisy on consumers, the influencing factors of consumers’ perception of hypocrisy, and the response of consumers to corporate hypocrisy. However, no study has indicated the measures to be adopted to address a corporate hypocrisy crisis with the aim of reducing the negative impact of the crisis. On the basis of existing research, this study investigated in detail the measures that enterprises should adopt to reduce the negative impact of a corporate hypocrisy crisis. This study also examined the features of hypocrisy manifestation (moral hypocrisy vs. behavioral hypocrisy) and its influence on consumer behavior Wagner et al. (2020). Few studies have classified corporate hypocrisy, and the ones that did so had certain limitations. The classification of corporate hypocrisy presented in this paper provides new research directions for better understanding corporate hypocrisy and adopting effective measures to handle a corporate hypocrisy crisis.
2. This study enriches the literature on corporate crisis management by investigating how enterprises should handle a corporate hypocrisy crisis. Studies on corporate crisis response have mainly focused on product harm, brand, and public opinion crises. Considerable differences exist in the characteristics of crises caused by corporate hypocrisy and the aforementioned three types of crises. Studies on corporate hypocrisy and corporate crisis response have not indicated how an enterprise should handle a crisis caused by consumers perceiving its CSR activities to be hypocritical. Consumers’ perception of hypocrisy in CSR activities might have an extremely negative effect on enterprises and cause them to experience a serious business crisis. This study suggests how enterprises should respond to a hypocrisy crisis.
3. This study introduces a new theoretical perspective based on SCCT for the examination of corporate hypocrisy. Most studies on corporate hypocrisy have examined the consumer response to corporate hypocrisy from the theoretical perspectives of attribution theory (Wang and Zhu, 2020), expectation theory (Wang et al., 2020), and identity theory (Miao and Zhou, 2020). On the basis of previous research, this study used SCCT to explore appropriate methods for handling a corporate hypocrisy crisis. On the basis of SCCT, this study proposes that different countermeasures should be adopted for different types of corporate hypocrisy to minimize the adverse impact of corporate hypocrisy. The introduction of SCCT into corporate hypocrisy research enriches the theoretical basis of such research.
4. This study expands the application scope of SCCT and enriches this theory. SCCT has been applied for examining various types of crises, such as product harm crises (Chen et al., 2019), network public opinion crises (Tang and Lai, 2017), overseas corporate crises (Lin and Liu, 2015), and doping crises in sport (Zhou and Wan, 2020). In the theoretical model of SCCT, crisis situation, crisis response strategy, historical corporate reputation, emotional response, and behavior tendency are crucial factors influencing crisis response. This study comprehensively considered the aforementioned influencing factors. Moral and behavioral hypocrisy crises can be divided into different categories according to their features. A reactive CSR communication strategy involves engaging in CSR-related communication after a crisis, whereas a proactive CSR communication strategy involves engaging in CSR-related communication for maintaining the reputation of an enterprise before the occurrence of a crisis. Negative emotions and negative behaviors represent the emotional response and behavior tendency, respectively. This study identified some additional crucial variables for SCCT in the context of corporate hypocrisy crises, including the type of crisis situation, the type of crisis response strategy, and compatibility between the crisis situation and crisis response strategy.

### Management Implications

This study explored the effects of corporate response strategies on consumers’ perception of hypocrisy in CSR activities. The results of this study can serve as a reference for enterprises to cope with different hypocrisy manifestation crises when consumers perceive hypocrisy in CSR implementation. An enterprise can adopt a suitable response strategy as per the findings of this study to reduce the adverse impact of a hypocrisy crisis on it and to regain confidence in CSR investment. The management implications of this study are as follows:

1. Enterprises must adopt an appropriate CSR communication strategy according to the type of manifestation of corporate hypocrisy. Different CSR communication strategies have distinct effects in different situations. Specifically, when a hypocrisy crisis originates from the immoral behavior of an enterprise, consumers regard the enterprise as being responsible for the crisis. Consequently, the enterprise is condemned at the moral level. In the aforementioned situation, the enterprise should adopt a reactive CSR communication strategy to alleviate the external pressure on it and attempt to correct its mistake for reducing the negative impact caused by the crisis. A proactive CSR communication strategy would not reduce the negative impact of the crisis in the aforementioned situation. When a hypocrisy crisis arises from the inconsistency between the words and deeds of an enterprise, consumers consider the hypocrisy to be unintentional; thus, the enterprise is not morally condemned by the public and bears limited responsibility for the crisis. In this situation, the enterprise can reduce the negative impact of the crisis on it to a greater extent by cultivating a good image through proactive CSR communication than through reactive CSR communication.
2. Enterprises must understand the role of consumers’ negative emotions in hypocrisy crisis events, and take measures to eliminate these emotions to avoid consumers’ negative behaviors. When a moral hypocrisy crisis occurs, enterprises should adopt a reactive CSR communication strategy to reduce consumers’ negative emotions. When a behavioral hypocrisy crisis occurs, cultivating a positive social image through proactive CSR communication would reduce consumers’ negative emotions to a greater extent than would engaging in reactive CSR communication.
3. Enterprises should avoid the occurrence of moral hypocrisy. The results of this study indicate that consumers’ perception of corporate moral hypocrisy produces more negative emotions among and more negative behaviors by them than does the perception of corporate behavioral hypocrisy. Enterprises should avoid immoral behavior and achieve consistency between their behavior and promised moral standards so that consumers would not perceive them to be engaged in moral hypocrisy. Consumers’ response to hypocrisy events is mainly governed by their perceptions (Wry, 2009); therefore, enterprises should adopt certain measures to guide consumers’ judgment and avoid consumers’ awareness of moral hypocrisy. The aforementioned goals can be achieved through two approaches. First, an enterprise can indicate the uncontrollable factors of a crisis event to consumers, thereby conveying a signal that the enterprise is a victim of the crisis. Such a strategy can help enterprises win the sympathy and understanding of consumers. Second, enterprises should avoid the transmission of self-interest-driven signals to consumers and prevent potential interest-related information from becoming the focus of consumers’ attention.
4. Enterprises should strive to control the occurrence of behavioral hypocrisy. Enterprises should conduct real-time evaluation and monitoring of the implementation process and effect of their CSR activities as well as predict and control factors that might affect CSR implementation. Moreover, enterprises should avoid promising CSR investment that would be difficult to accomplish. Enterprises should avoid inconsistency between their words and deeds through the aforementioned measures. Although an enterprise would not be morally condemned for the occurrence of such inconsistency, these inconsistency would result in the enterprise being considered unreliable and unpredictable. Such a perception by consumers would have a negative impact on the enterprise. In the aforementioned scenario, if the enterprise does not engage in long-term CSR investment in the relevant field, it would find it difficult to reduce the negative impact of the aforementioned perception. However, if the enterprise engages in long-term CSR investment, it may incur an extremely high cost. If CSR-related remedial behavior is conducted in the aforementioned situation, consumers would doubt the CSR motivation. In this scenario, consumers’ negative perception of the enterprise might be related to moral factors.
5. Enterprises should actively undertake social responsibility and strive to establish a positive social image of themselves in the minds of stakeholders. When a hypocrisy crisis occurs, the positive image established by an enterprise through active participation in CSR activities may prove beneficial as the company attempts to resolve the crisis. This statement is valid only when enterprises avoid immoral behavior. If enterprises engage in immoral behavior, their social image would be ruined. Therefore, actively taking social responsibility and acting according to moral standards are indispensable steps for developing a good social image for an enterprise.

### Limitations and Directions for Future Research

Through a scenario simulation experiment, this study examined response strategies of enterprises for corporate hypocrisy crises. The results of this study indicate that different CSR communication strategies have different effects under different types of hypocrisy crises. The results also indicate the influence of the response strategies adopted by enterprises on consumer behavior in different scenarios. Although this study makes theoretical and practical contributions, it has certain limitations, which must be addressed by future studies.

First, this study conducted a scenario simulation experiment and collected questionnaire data. The consumer responses obtained in such a manner are susceptible to social desirability bias and might be different from consumers’ true responses. Future studies can examine corporate hypocrisy by collecting consumer data in a real scene to improve the external validity of the research. Second, this study selected negative emotions as the mediator variable to analyze the influence of response strategies for corporate hypocrisy on consumer behavior. However, in addition to negative emotions, other feelings, such as trust, betrayal, identity, and doubt, have crucial influences on consumer behavior. Future studies can explore the effects of these factors on consumers’ reaction to response strategies for a corporate hypocrisy crisis. Third, this study classified corporate hypocrisy from the perspective of hypocrisy manifestation. Other methods exist for classifying corporate hypocrisy, such as classification based on the causes and consequences of hypocrisy. Future studies can classify corporate hypocrisy by using different methods and determine suitable response strategies for corporate hypocrisy on the basis of these classifications.

## Data Availability Statement

The original contributions presented in the study are included in the article/supplementary material, further inquiries can be directed to the corresponding authors.

## Ethics Statement

Ethical review and approval was not required for the study on human participants in accordance with the local legislation and institutional requirements. Written informed consent from the patients/participants was not required to participate in this study in accordance with the national legislation and the institutional requirements.

## Author Contributions

All authors listed have made a substantial, direct, and intellectual contribution to the work and approved it for publication.

## Funding

This study was supported by young and middle aged scientific research team of Wuhan Sports University in 2021 (21KT18), philosophy and social science research project of Hubei Education Department (19Y099), scientific research project of Hubei Education Department (B2021186), and Youth Scientific Research Fund of Wuhan Sports University (2022S05).

## Conflict of Interest

RL is employed by China Baowu Iron and Steel Group Co., Ltd.

The remaining authors declare that the research was conducted in the absence of any commercial or financial relationships that could be construed as a potential conflict of interest.

## Publisher’s Note

All claims expressed in this article are solely those of the authors and do not necessarily represent those of their affiliated organizations, or those of the publisher, the editors and the reviewers. Any product that may be evaluated in this article, or claim that may be made by its manufacturer, is not guaranteed or endorsed by the publisher.
